# Pacritinib‐Based Salvage Therapy in High‐Risk Polycythaemia Vera With Recurrent Arterial and Venous Thrombosis After Hydroxyurea and Ruxolitinib: A Case Report

**DOI:** 10.1155/crh/8282682

**Published:** 2026-10-16

**Authors:** Muna Al-Rasheed

**Affiliations:** ^1^ Haematology Unit Internal Medicine Department Adan Hospital MOH Hadiya Kuwait

**Keywords:** JAK inhibitors, pacritinib, polycythaemia vera, thrombosis

## Abstract

**Objectives:**

Polycythaemia vera (PV) is associated with substantial arterial and venous thrombotic risk, particularly in patients with prior thrombosis and inadequate haematocrit (HCT) control. Although ruxolitinib is an established second‐line treatment after hydroxyurea failure or intolerance, some patients remain difficult to manage. We report a case of highly thrombotic, treatment‐challenging PV in which pacritinib‐based salvage therapy was used after inadequate disease control with hydroxyurea and ruxolitinib.

**Case Presentation:**

A 44‐year‐old man was referred with portal vein thrombosis and a history of premature myocardial infarction and cerebrovascular accident. Investigations demonstrated JAK2V617F‐positive PV with borderline low erythropoietin and bone marrow panmyelosis. Despite antiplatelet therapy, anticoagulation, hydroxyurea and subsequent ruxolitinib, the patient experienced further thrombotic complications, including recurrent cerebrovascular events, bilateral limb ischaemia and left ventricular thrombosis. Ruxolitinib was escalated to 25 mg twice daily and combined with low‐dose hydroxyurea; however, HCT control remained suboptimal, with recurrent need for venesection. In February 2025, ruxolitinib was replaced with pacritinib 200 mg twice daily while hydroxyurea and warfarin were continued. During follow‐up, the patient achieved improved HCT control, no longer required venesection and had no further documented thrombotic events. Pacritinib was well tolerated.

**Conclusion:**

This case describes a patient with high‐risk, refractory PV and recurrent arterial and venous thrombosis in whom pacritinib‐based salvage therapy was associated with improved HCT control, cessation of venesection and absence of further thrombosis during follow‐up. Although off‐label and hypothesis‐generating, this experience supports further evaluation of pacritinib in selected patients with treatment‐challenging PV.

## 1. Introduction

Polycythaemia vera (PV) is a chronic, myeloproliferative neoplasm (MPN) characterised by clonal erythrocytosis, substantial thrombohaemorrhagic risk, symptom burden and potential progression to post‐PV myelofibrosis or acute myeloid leukaemia [1]. The disease is driven in most cases by aberrant Janus kinase–signal transducer and activator of transcription (JAK‐STAT) signalling, most commonly through JAK2 alterations [2]. In contemporary practice, the principal therapeutic objective in PV remains prevention of thrombosis, with conventional risk stratification based primarily on age older than 60 years and prior thrombotic history. Haematocrit (HCT) control below 45% has been associated with a significantly lower risk of cardiovascular death and major thrombosis [3]. Current treatment algorithms for PV combine phlebotomy and low‐dose aspirin for most patients, with cytoreductive therapy recommended for those at high thrombotic risk, particularly patients with previous thrombosis [1].

However, durable disease control is not universal, and a clinically meaningful proportion of patients develop hydroxyurea resistance, intolerance or persistent proliferative activity despite treatment [4, 5]. Ruxolitinib has become the established second‐line therapeutic standard for adults with PV after inadequate response to or intolerance of hydroxyurea [1]. In the pivotal RESPONSE study (NCT01243944), ruxolitinib was superior to standard therapy in achieving HCT control and spleen response [6], while RESPONSE‐2 (NCT02038036) extended this benefit to patients without splenomegaly and supported durable second‐line use over longer follow‐up [7]. Nevertheless, a subset of patients remains difficult to manage because of recurrent thrombosis, persistent need for venesection, incomplete haematological control, limited access to interferon or failure of sequential standard approaches [8].

Pacritinib is a mechanistically distinct, JAK1‐sparing kinase inhibitor with activity against JAK2, mutant JAK2V617F, interleukin‐1 receptor‐associated kinase 1 (IRAK1) and activin A receptor type 1 (ACVR1) [9]. In the phase 3 PERSIST‐1 trial (NCT01773187), pacritinib 400 mg once daily demonstrated superior spleen responses versus best available therapy, with week‐24 spleen volume reduction (SVR) ≥ 35% achieved in 19% versus 5% of the patients, including activity in those with severe thrombocytopenia [10]. In the Phase 3 PERSIST‐2 trial (NCT02055781), pacritinib 200 mg twice daily was superior to best available therapy, including ruxolitinib, for both spleen and symptom endpoints, with SVR ≥ 35% in 22% versus 3% and ≥ 50% reduction in total symptom score in 32% versus 14%; among patients with baseline platelet counts < 50 × 10^9^/L, SVR ≥ 35% was 29% versus 3.1% [11]. The subsequent PAC203 study (NCT03165734) further supported 200 mg twice daily as the preferred dose in advanced, ruxolitinib‐exposed myelofibrosis [12]. Although pacritinib is not a standard therapy for PV, and evidence in this setting remains extremely limited, these results provide a clinically relevant rationale for reporting carefully documented off‐label experience in highly selected patients with persistent disease activity despite conventional and second‐line treatment.

We present the case of a patient with high‐risk PV complicated by recurrent arterial and venous thrombosis, including portal vein thrombosis, with persistent therapeutic challenges despite hydroxyurea, anticoagulation and ruxolitinib, who subsequently underwent treatment with pacritinib.

## 2. Case Presentation

This manuscript was prepared in accordance with the CARE checklist. Written informed consent has been obtained from the patient to publish this paper. The patient was assessed and managed at the Haematology Unit, Internal Medicine Department, Adan Hospital, Ministry of Health, Kuwait, between January 2021 and December 2025.

A 44‐year‐old man was referred to the haematology clinic in January 2021 after presenting with portal vein thrombosis. His past medical history was notable for an acute myocardial infarction at the age of 30 years and a cerebrovascular accident at the age of 33 years, both reportedly occurring without identified conventional risk factors and without prior comprehensive haematological investigation. Following the cerebrovascular event, he had been treated with aspirin and clopidogrel. At the time of referral, the complete blood count (CBC) showed haemoglobin (Hb) 160 g/L, HCT 46%, white blood cell (WBC) count 7.6 × 10^9^/L, and platelet count 430 × 10^9^/L. Further evaluation showed negative inherited thrombophilia screening, negative antiphospholipid antibody testing, and negative flow cytometry for paroxysmal nocturnal haemoglobinuria. Antiphospholipid antibody testing and paroxysmal nocturnal haemoglobinuria screening were repeated following the subsequent recurrent thrombotic events and remained negative on both occasions. Molecular testing demonstrated JAK2V617F positivity, serum erythropoietin was borderline low and bone marrow biopsy showed panmyelosis with grade 1/4 reticulin fibrosis. On this basis, the patient was diagnosed with high‐risk PV in view of recurrent arterial and venous thrombosis. No alternative inherited or acquired prothrombotic disorder was identified in the available investigations.

Following diagnosis, aspirin and clopidogrel were continued after discussion with cardiology and neurology, and direct oral anticoagulant (DOAC) therapy was initiated immediately with a loading regimen followed by maintenance treatment. Cytoreductive therapy was commenced with hydroxyurea at an initial dose of 500 mg twice daily. During this phase, the patient had no major adverse effects and did not require venesection. HCT remained below the recommended 45% threshold, with a mean of 40% and a range of 38%–43%, indicating adequate early HCT control.

In 2023, the patient was admitted with a new ischaemic cerebrovascular accident complicated by haemorrhagic transformation. In view of the intracranial bleeding, DOAC, aspirin and clopidogrel were discontinued, with a plan to transition to warfarin once clinically stable. In parallel, the PV was considered inadequately controlled on hydroxyurea, and second‐line therapy with ruxolitinib was initiated at 10 mg twice daily for 2 years. During the interval off anticoagulation, the patient developed further extensive thrombosis, including bilateral lower‐limb ischaemia, bilateral upper‐limb ischaemia requiring surgical intervention and left ventricular thrombosis. Anticoagulation was, therefore, restarted using heparin infusion. Repeat computed tomography of the head was performed to confirm no progression of the intracranial bleed, after which warfarin was added with a target international normalised ratio (INR) of 2.0–3.0.

The patient remained clinically stable on warfarin. However, haematological control became suboptimal despite cytoreductive therapy, and three venesections were required. Ruxolitinib was escalated to 25 mg twice daily in July 2024. Because HCT control remained suboptimal, hydroxyurea 500 mg once daily was subsequently reintroduced in combination with ruxolitinib. Despite this regimen, HCT remained approximately 45%–50%, and venesection was required approximately once monthly. Further hydroxyurea dose escalation was not pursued because hydroxyurea had previously provided inadequate disease control and additional dose intensification in combination with high‐dose ruxolitinib was considered unlikely to provide durable control while potentially increasing myelosuppressive toxicity. Serial laboratory findings demonstrated persistent Hb values of approximately 146–155 g/L, with no new thrombotic or bleeding events. During this period, warfarin monitoring remained largely within or near target, with INR values ranging from 2.12 to 2.54. These findings are consistent with persistent erythrocytosis despite ongoing therapy.

Given the persistent need for venesection despite combination cytoreductive therapy, ruxolitinib was subsequently replaced by pacritinib 200 mg twice daily in February 2025, while hydroxyurea 500 mg once daily was continued. Prior to pacritinib initiation, laboratory findings showed WBC 6.2 × 10^9^/L, red blood cell (RBC) count 4.72 × 10^12^/L, Hb 136 g/L, HCT 47.6%, and platelets 236 × 10^9^/L, with INR 2.16. Early post‐switch monitoring showed WBC count 6.3 × 10^9^/L, Hb 142 g/L, HCT 42.4%, and platelets 154 × 10^9^/L. A subsequent assessment in early April 2025 showed a transient increase in HCT to 49.3%; repeat testing approximately one week later demonstrated spontaneous reduction to approximately 42% without venesection or modification of cytoreductive therapy. HCT remained below 45% thereafter, including 43.0% in May 2025 and 41.1% at the final follow‐up in December 2025 (Figures 1 and 2).

**FIGURE 1:**
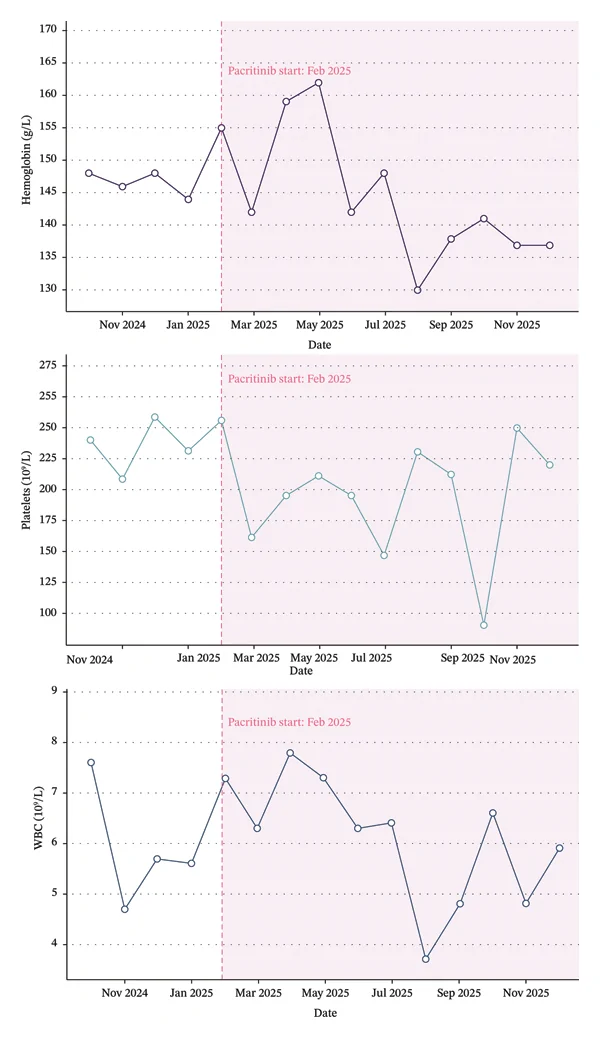
Longitudinal changes in haemoglobin, platelet count and white blood cells before and after pacritinib treatment. The vertical dashed line indicates initiation of pacritinib in February 2025; the shaded area denotes the pacritinib treatment period.

**FIGURE 2:**
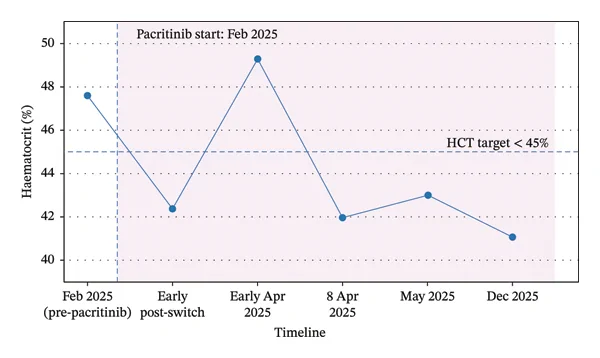
Longitudinal changes in haematocrit before and after the initiation of pacritinib. The vertical dashed line indicates initiation of pacritinib in February 2025. The horizontal dashed line represents the recommended HCT target of < 45%. No venesection was required after pacritinib initiation.

Further follow‐up demonstrated relative stabilisation of the haematological profile till December 2025. At the last follow‐up, Hb was 137 g/L, HCT was 41.1%, and platelets were 205 × 10^9^/L. INR values during follow‐up were generally maintained in or near the target range, mostly between 2.31 and 3.23. The patient no longer required venesection, and no further thrombotic events were reported. Pacritinib 200 mg was well tolerated, and no further thrombotic manifestations were observed during the follow‐up period.

## 3. Discussion

This case illustrates several clinically important themes in the management of PV. First, it highlights an unusually thrombotic disease phenotype, with arterial and venous events occurring over time, including myocardial infarction, cerebrovascular events, portal vein thrombosis, limb ischaemia, and left ventricular thrombosis. Second, it underscores the challenge of maintaining durable haematological control in a patient with recurrent thrombosis despite the sequential use of anticoagulation, hydroxyurea and maximally escalated ruxolitinib. Third, it raises the possibility that pacritinib‐based salvage therapy may have clinical utility in highly selected patients with persistently active, high‐risk PV after failure of conventional and second‐line approaches. In the present case, the switch from ruxolitinib to pacritinib, with continuation of low‐dose hydroxyurea and warfarin, was followed by cessation of venesection, improvement in HCT control and absence of further documented thrombosis during follow‐up.

An important aspect of this case is the initial presentation of the patient. Portal vein thrombosis is a recognised manifestation of MPNs, and MPNs are among the commonest underlying causes of primary splanchnic vein thrombosis [13, 14]. In this setting, diagnosis can be challenging because classical features of PV may be attenuated or obscured by portal hypertension, haemodilution, iron deficiency or prior bleeding [15]. Recent data in JAK2‐mutated splanchnic thrombosis suggest that a substantial proportion of such patients meet criteria for masked PV when red cell mass or marrow findings are considered [13]. The present patient is clinically consistent with this diagnostic paradigm: despite only modestly abnormal indices at referral, the combination of JAK2 positivity, borderline low erythropoietin, marrow panmyelosis and an unusual‐site thrombotic event supported the diagnosis of PV.

The case also reinforces the central importance of thrombotic prevention in PV. Contemporary management is primarily directed at reducing vascular events, with prior thrombosis placing patients in the high‐risk category irrespective of age. In CYTO‐PV, maintaining HCT below 45% significantly reduced the rate of cardiovascular death and major thrombosis compared with less stringent control [3]. This threshold remains a cornerstone of modern management recommendations. In the present case, recurrent need for venesection and repeated periods of HCT in the 45%–50% range while on ruxolitinib‐based therapy were, therefore, clinically meaningful rather than simply laboratory observations because they implied ongoing exposure to a validated thrombotic risk factor in a patient who had already declared an extreme vascular phenotype.

Ruxolitinib is the accepted second‐line standard for PV after inadequate response to or intolerance of hydroxyurea. In the pivotal RESPONSE study, ruxolitinib was superior to standard therapy for the composite endpoint of HCT control without phlebotomy and SVR, while also improving symptom burden. RESPONSE‐2 extended the benefit of ruxolitinib to patients without splenomegaly and confirmed more effective HCT control in this population. These data established ruxolitinib as the evidence‐based option after hydroxyurea failure; nonetheless, they do not imply that all patients will achieve durable or complete disease control. The present case exemplifies such a scenario: despite escalation to 25 mg twice daily and subsequent reintroduction of hydroxyurea, the patient continued to require venesection and remained difficult to control haematologically, even though no further overt thrombosis or bleeding was documented during that phase.

The most notable feature of this case is the apparent improvement in HCT control after switching from ruxolitinib to pacritinib, despite ruxolitinib being the approved JAK inhibitor for hydroxyurea‐resistant or ‐intolerant PV and having demonstrated superior HCT control in randomised PV studies in randomized PV studies [6]. This observation is, therefore, hypothesis‐generating rather than practice‐defining, but it raises an important mechanistic question: whether pacritinib may have provided disease control through kinase effects not shared by ruxolitinib. Ruxolitinib inhibits JAK1 and JAK2, whereas pacritinib is relatively JAK1‐sparing and inhibits JAK2, IRAK1, FMS‐like tyrosine kinase 3 (FLT3) and colony‐stimulating factor 1 receptor (CSF1R), with later work also demonstrating potent ACVR1 inhibition [16].

At first glance, pacritinib‐associated HCT control is counterintuitive, as its ACVR1 activity has been linked to improvement in inflammatory anaemia in myelofibrosis through suppression of hepcidin and increased iron availability [9]. In the present case, however, the clinical problem was erythrocytosis rather than inflammatory anaemia. Thus, ACVR1 inhibition is unlikely to explain HCT control and could, in theory, support rather than suppress erythropoiesis. The transient postswitch rise in Hb/HCT observed 2 months after treatment is consistent with this uncertainty and argues against ACVR1 as the central mechanism in this patient.

A more plausible hypothesis relates to IRAK1 inhibition. IRAK1 participates in interleukin‐1 (IL‐1)/Toll‐like receptor signalling and downstream nuclear factor kappa B (NF‐κB)–mediated inflammatory cytokine signalling [17]. Inflammation is increasingly recognized as a contributor to MPN biology beyond canonical JAK‐STAT activation, and preclinical JAK2V617F models suggest that IL‐1 signalling can promote clonal expansion, abnormal blood counts, splenomegaly and marrow fibrosis [17, 18]. Therefore, dual JAK2/IRAK1 inhibition could, in principle, suppress both JAK2‐driven erythroid proliferation and inflammatory pathways that reinforce the malignant clone or its haematopoietic niche. This mechanism remains speculative in PV, but among pacritinib’s nonruxolitinib targets, IRAK1 is the most biologically coherent candidate for explaining a differential effect on disease control.

By contrast, FLT3 inhibition is a less compelling explanation, as FLT3 is not known to be a dominant driver of erythroid overproduction in PV. CSF1R inhibition could theoretically influence monocyte/macrophage biology, inflammatory signalling, splenic haematopoiesis or iron handling, but direct evidence linking CSF1R inhibition to HCT control in PV is limited. Thus, if pacritinib exerted a unique effect in this case, it was more likely mediated through combined JAK2 and inflammatory‐pathway inhibition, particularly IRAK1, rather than through FLT3 or ACVR1.

Several nonmutually exclusive clinical explanations should also be acknowledged. First, hydroxyurea was continued after pacritinib initiation, so the subsequent HCT improvement cannot be attributed to pacritinib alone. Second, ruxolitinib failure in an individual patient does not necessarily imply class failure because response may be affected by dose intensity, treatment duration, adherence, phlebotomy requirements, iron status, intercurrent inflammation and disease kinetics. Third, the observation is based on a single case without paired cytokine profiling, JAK2V617F allele burden monitoring, hepcidin levels or erythroid progenitor studies. Accordingly, this case should be interpreted as evidence of a possible clinical signal rather than proof of a pacritinib‐specific mechanism.

Against this background, the rationale for pacritinib in this case was biologically plausible but clinically off‐label. Pacritinib is not approved for PV; its current regulatory indication is intermediate‐ or high‐risk primary or secondary myelofibrosis, including post‐PV or post‐essential thrombocythaemia (ET) myelofibrosis, in adults with platelet counts below 50 × 10^9^/L.

## 4. Conclusion

This case describes a patient with highly thrombotic, treatment‐challenging PV in whom pacritinib‐based salvage therapy was followed by improved haematological control and no further documented thrombotic events during follow‐up. This limited real‐world experience supports further evaluation of pacritinib in selected patients with refractory PV.

## Author Contributions

Muna Al‐Rasheed conceived the report, provided clinical care to the patient, collected and interpreted the clinical data and critically reviewed and revised the manuscript.

## Funding

Sobi provided funding for the medical writing activities and open‐access fees of this manuscript through a medical grant but did not influence the content or direction of the manuscript and was allowed a courtesy review.

## Disclosure

Muna Al‐Rasheed had full access to all of the data in this study and takes complete responsibility for the integrity of the data and the accuracy of the data analysis. The author approved the final version for submission.

## Ethics Statement

This is a single‐patient case report; institutional review board approval was not required at Adan Hospital, Ministry of Health, Kuwait. The report was conducted in accordance with the Declaration of Helsinki.

## Consent

Written informed consent for publication of this case report and any accompanying data was obtained from the patient. A copy of the signed consent form is available to the editorial office on request.

## Conflicts of Interest

The author declares no conflicts of interest.

## Supporting Information

Additional supporting information can be found online in the Supporting Information section.

## Supporting information


**Supporting Information** CARE‐checklist‐English‐2013

## Data Availability

The data supporting the findings of this case report are available from the corresponding author upon reasonable request. The data are not publicly available because of patient privacy considerations.
