# Supplementary material for: Pacritinib‐Based Salvage Therapy in High‐Risk Polycythaemia Vera With Recurrent Arterial and Venous Thrombosis After Hydroxyurea and Ruxolitinib: A Case Report
Source: Case Rep Hematol. 2026 Oct 16;2026:8282682. doi: 10.1155/crh/8282682 (PMC13673302; doi:10.1155/crh/8282682)
Supplement: Supplementary file 1 [file CRH-2026-8282682-s001.pdf]

CARE
case report guidelines

CARE Checklist of information to include when writing a case report

(cc) BY-NC-ND

Topic	Item	Checklist item description	Reported on Line
**Title**	1	The diagnosis or intervention of primary focus followed by the words "case report"	1-3
**Key Words**	2	2 to 5 key words that identify diagnoses or interventions in this case report, including "case report"	38-39
**Abstract (no references)**	3a	Introduction: What is unique about this case and what does it add to the scientific literature?	16-22
	3b	Main symptoms and/or important clinical findings	23-29
	3c	The main diagnoses, therapeutic interventions, and outcomes	23-34
	3d	Conclusion—What is the main "take-away" lesson(s) from this case?	35-37
**Introduction**	4	One or two paragraphs summarizing why this case is unique (**may include references**)	40-81
**Patient Information**	5a	De-identified patient specific information.	87-88
	5b	Primary concerns and symptoms of the patient.	87-88
	5c	Medical, family, and psycho-social history including relevant genetic information	88-100
	5d	Relevant past interventions with outcomes	91-106
**Clinical Findings**	6	Describe significant physical examination (PE) and important clinical findings.	87-102
**Timeline**	7	Historical and current information from this episode of care organized as a timeline	87-159; Figs 1-2
**Diagnostic Assessment**	8a	Diagnostic testing (such as PE, laboratory testing, imaging, surveys).	92-101, 111-116
	8b	Diagnostic challenges (such as access to testing, financial, or cultural)	NR
	8c	Diagnosis (including other diagnoses considered)	92-102
	8d	Prognosis (such as staging in oncology) where applicable	101-102
**Therapeutic Intervention**	9a	Types of therapeutic intervention (such as pharmacologic, surgical, preventive, self-care)	103-137
	9b	Administration of therapeutic intervention (such as dosage, strength, duration)	103-137
	9c	Changes in therapeutic intervention (with rationale)	107-137
**Follow-up and Outcomes**	10a	Clinician and patient-assessed outcomes (if available)	138-143, 154-159
	10b	Important follow-up diagnostic and other test results	136-143, 154-158
	10c	Intervention adherence and tolerability (How was this assessed?)	158-159
	10d	Adverse and unanticipated events	104-115, 158-159
**Discussion**	11a	A scientific discussion of the strengths AND limitations associated with this case report	160-169, 239-249
	11b	Discussion of the relevant medical literature **with references**.	160-238
	11c	The scientific rationale for any conclusions (including assessment of possible causes)	189-249
	11d	The primary "take-away" lessons of this case report (without references) in a one paragraph conclusion	255-258
**Patient Perspective**	12	The patient should share their perspective in one to two paragraphs on the treatment(s) they received	NR
**Informed Consent**	13	Did the patient give informed consent? Please provide if requested	**Yes** ☑ **No** ☐
